# *In vitro* systems to study inborn errors of immunity using human induced pluripotent stem cells

**DOI:** 10.3389/fimmu.2022.1024935

**Published:** 2022-11-17

**Authors:** Eirini Nikolouli, Janne Reichstein, Gesine Hansen, Nico Lachmann

**Affiliations:** ^1^ Department for Pediatric Pneumology, Allergology and Neonatology, Hannover Medical School (MHH), Hannover, Germany; ^2^ Cluster of Excellence - Resolving Infection Susceptibility (RESIST, EXC 2155), Hannover Medical School, Hannover, Germany; ^3^ Biomedical Research in Endstage and Obstructive Lung Disease Hannover (BREATH), German Center for Lung Research (DZL), Hannover, Germany; ^4^ Regenerative Biology to Reconstructive Therapy (REBIRTH) Center for Translational and Regenerative Medicine, Hannover Medical School, Hannover, Germany

**Keywords:** inborn errors of immunity, iPSCs, disease modeling, cell therapies, immune cells, macrophages

## Abstract

In the last two decades, the exponential progress in the field of genetics could reveal the genetic impact on the onset and progression of several diseases affecting the immune system. This knowledge has led to the discovery of more than 400 monogenic germline mutations, also known as “inborn errors of immunity (IEI)”. Given the rarity of various IEI and the clinical diversity as well as the limited available patients’ material, the continuous development of novel cell-based *in vitro* models to elucidate the cellular and molecular mechanisms involved in the pathogenesis of these diseases is imperative. Focusing on stem cell technologies, this review aims to provide an overview of the current available *in vitro* models used to study IEI and which could lay the foundation for new therapeutic approaches. We elaborate in particular on the use of induced pluripotent stem cell-based systems and their broad application in studying IEI by establishing also novel infection culture models. The review will critically discuss the current limitations or gaps in the field of stem cell technology as well as the future perspectives from the use of these cell culture systems.

## Introduction

In the last decades, the major progress in the field of genetics and the availability of high-throughput DNA sequencing techniques contributed to the discovery of more than 400 monogenic germline mutations affecting our immune system. These mutations are referred to as inborn errors of immunity (IEI) and can lead either to the loss of expression or loss/gain of function of the respective protein (1–3). The prevalence of IEI in the overall population is in the range of 1/10,000-1/50,000 (4). In most cases, IEI are identified early in life upon recurring infections such as bronchitis or sinusitis and can be life-threatening if the patients do not receive proper treatment. The clinical phenotype of IEI shows a variety of disorders, including autoimmune or inflammatory diseases, allergies, cancer, and increased susceptibility to several pathogens.

According to the International Union of Immunological Societies, the IEI are classified into the following ten categories of conditions: Combined immunodeficiencies; Combined immunodeficiencies with syndromic features; Predominantly antibody deficiencies; Diseases of immune dysregulation; Congenital defects of phagocytes; Defects in intrinsic and innate immunity; Autoinflammatory diseases; Complement deficiencies, Bone marrow failure, and Phenocopies of IEI (2–5).

Due to the variable clinical features of IEI-related disorders, the medical care and treatment of these young patients is extremely challenging and requires a careful fine-tuning of the immune system. Children with IEI are usually treated with immunosuppressants, such as rapamycin or corticosteroids to decrease inflammation, however, this leads to a broad range of side effects. In addition, these types of treatment can only alleviate the symptoms but do not offer a curative solution for the patient. Other therapeutic strategies include the long-term usage of anti-fungal, anti-viral, or anti-bacterial agents, increasing the risk for the development of drug-resistant pathogens, which can cause life-threatening infections. In some cases, like in the severe combined immunodeficiency (SCID) syndrome, allogenic hematopoietic stem cell transplantation (HSCT) (or autologous HSC-gene therapy) is the only curative therapy (6, 7). However, HSCT always lurks the risk of immunological rejection or development of graft versus host disease with devastating consequences for the patient, pointing towards the need of suitable alternatives.

For these reasons, more targeted therapeutic approaches, which can directly modulate specific cell types or intracellular pathways, are preferred. These approaches include the use of specific inhibitors or biologics (antibodies or recombinant proteins). For the safe use of these emerging therapeutic agents, a detailed study of the pathophysiological mechanisms of the diseases is necessary. Given the rarity of IEI and the technical difficulties (obtaining sufficient samples from children or the low number of affected cells), the study of IEI-related diseases remains challenging. Thus, the development of novel systems to unravel the cellular and molecular mechanisms involved in the pathophysiology of the various IEI is of great importance.

## Cell-based *in vitro* systems to study IEI

In the last years, the establishment of novel IEI *in vitro* systems has contributed enormously to the current understanding of the immunopathology involved in the various clinical features of different IEI-related diseases. As a consequence, these insights allowed for the development of new therapeutic approaches. The most appropriate *in vitro* models developed for these purposes are stem-cell based since stem cells have the capacity for self-renewal and differentiation into specialized cell types. The two main approaches used are based either on adult hematopoietic stem cells (HSCs) or induced pluripotent stem cells (iPSCs).

Adult HSCs are primary cells isolated from different sources such as peripheral blood, bone marrow, or umbilical cord. Their low number, inefficient long-term expansion, and heterogeneity however, impact their use in disease modeling and clinical applications.

**Figure 1:**
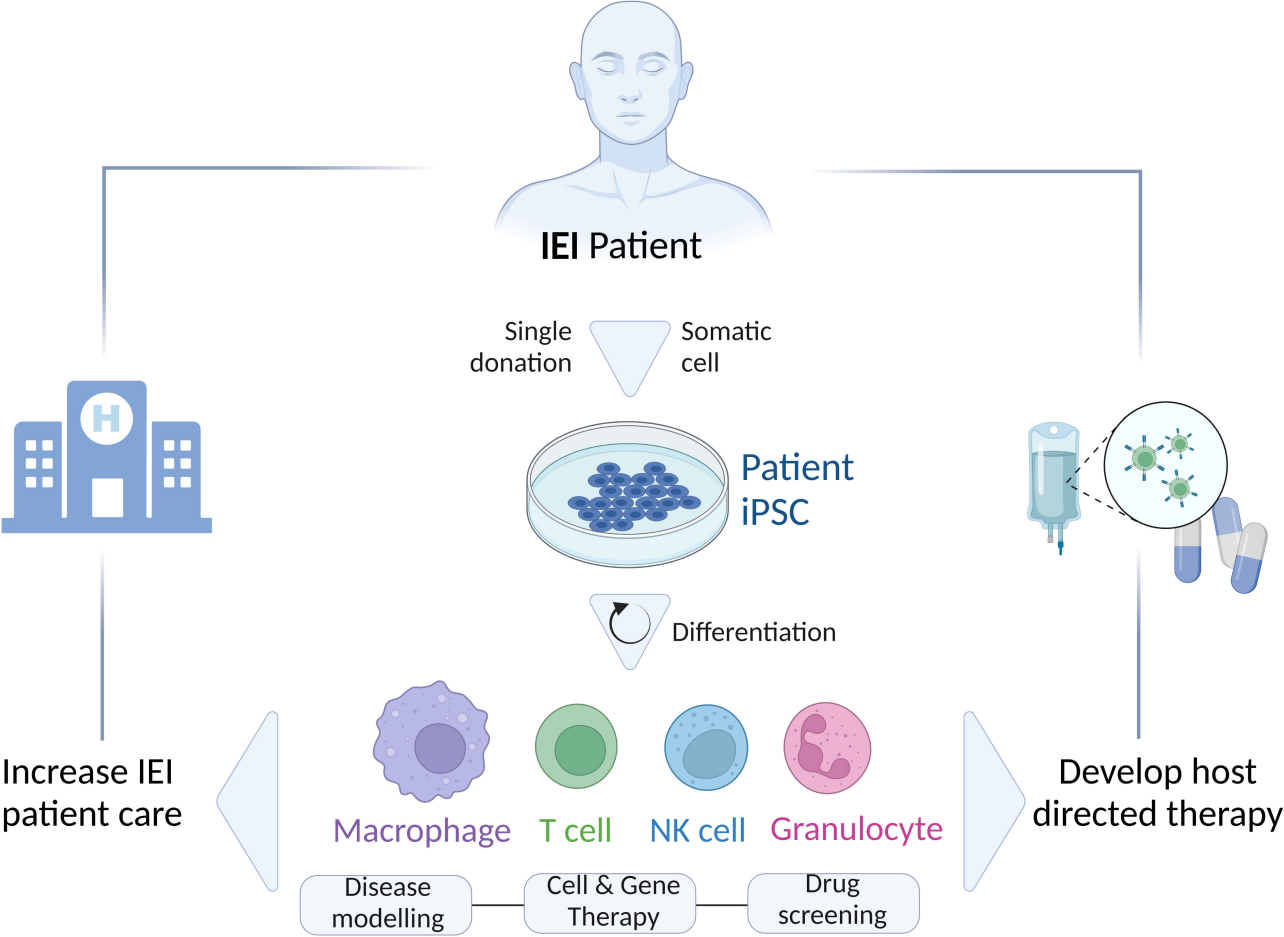
Schematic representation of the generation and differentiation of patient’s-specific iPSCs to different cell types (Macrophages, T cells, NK cells, Granulocytes) for various applications (Disease modeling; Cell and Gene therapy; Drug screening) to study IEI and improve the patient’s care by developing novel host directed therapies. IEI, inborn errors of immunity; iPSC, induced-pluripotent stem cells.

As an alternative, iPSC-based *in vitro* models have proven to be one of the most successful options to adequately study IEI (8). Reprogramming of a few somatic cells, isolated from a patient, leads to the generation of stable, pluripotent, and patient-specific iPSC lines, which can give rise indefinitely to various cell types (**Figure 1**). Thus, iPSC technology becomes a promising tool to investigate the possible mechanisms involved in the pathophysiology of IEI using various cell types generated through specialized differentiation protocols from a single iPSC line. In addition, the fact that the generation of iPSCs is based on less-invasive methods for the patient renders the iPSC-derived cells preferable in comparison to primary specialized cells isolated consecutively from patients with more laborious and invasive procedures. Of note, this plays a particular role when children are affected.

Studying the immune system using animal models has given us insights into its function. However, in cases of IEI, which affect hematopoiesis and immune development, the inter-species differences within hematopoietic development are a considerable limitation for the use of animal models to adequately study IEI (9). In those cases, human iPSC-derived cells are better suited to clarify the role of specific IEI in the hematopoietic system.

Furthermore, the use of iPSC-derived cells is not only advantageous for studying IEI to unravel the pathomechanism of various diseases but also introduces alternative therapeutic strategies. The phenotypical and functional similarities of iPSC-derived immune cells such as granulocytes, macrophages, and dendritic cells with their respective primary counterparts (10) further support the use of iPSC-derived cells to study the onset of diseases and to develop novel cell therapy concepts. As a consequence, in the last years continuous optimization of the iPSC-based differentiation protocols improved both the quality and quantity of the derived cell types, aiming to fulfill the requirements for clinical application.

## Hematopoietic differentiation protocols for the generation of iPSC-derived immune cells to study IEI

The fact that various tissues and cell types may be affected in different IEI underlines the complexity of IEI-related diseases and the growing need for specialized and highly standardized immune cells to study the onset and progression of these diseases. The high demand for adequate numbers of the affected patient-specific immune cells led, upon the discovery of iPSCs in 2006, to the establishment of numerous hematopoietic differentiation protocols able to generate different lineages of the lympho-hematopoietic system. In recent years, several iPSC-based differentiation protocols have been established for the generation of for instance macrophages, granulocytes, dendritic cells (DCs), natural killer cells (NK), NKT cells, and T lymphocytes. In general, the iPSC differentiation protocols include as first step the differentiation of iPSCs to hematopoietic progenitors either by the support of stromal cells and the use of cytokines or by the formation of so-called embryonic bodies (EBs), which are aggregates containing cells of the three germ layers. As an example, in the context of macrophages, recent differentiation techniques result in the generation of cells using EBs. The differentiation to hematopoietic or myeloid progenitors within the cell aggregates happens either autonomously from factors produced by the cell aggregates (11–13) or by the addition of exogenous factors such as BMP4, VEGF, SCF, Flt3-ligand, and TPO (13–15). Of note, using modern differentiation media authentic macrophages can be generated from human iPSCs, which share phenotypical and functional hallmarks with their *in vivo* counterparts (16). Similarly, simplified two-step protocols have also been established for the generation of iPSC-derived NK cells. In these differentiation platforms, the cytokines IL-3, IL-7, SCF, IL-15 and Flt3-ligand are often used for the differentiation of iPSC-derived progenitor cells towards NK cells (17, 18). Although still quite challenging to generate from human iPSC, some progress has been made to produce iPSC-derived T lymphocytes (19–28). Some of the strategies that have been developed include the reprogramming of antigen-specific T cells to iPSCs and the subsequent differentiation to T cells with the respective antigen-specificity (20, 23, 27) or the generation of custom-made antigen-specific T cells using T-cell receptor (TCR)-transduced iPSCs (22, 26). Of note, the functional resemblance of the iPSC-derived lymphocytes to the *in vivo* lymphocytes raises hopes for the use of these cells for the treatment of several diseases.

Moving from the use of iPSC-derived immune cells for disease modelling towards cell-based therapies targeting IEI, required the development of differentiation protocols which allow a continuous and scalable production of iPSC-derived immune cells such as macrophages (12, 29), NK cells (30), and T lymphocytes (22). The successful use of iPSC-derived immune cells as a cell-based therapy requires authentic immune cells, which are functionally indistinguishable from their *in vivo* counterparts. For instance, iPSC-derived macrophages show typical morphological and phenotypical characteristics. When tested for their ability to secrete cytokines and to perform phagocytosis, iPSC-macrophages showed a similar cytokine secretion profile to the monocyte-derived macrophages and high phagocytic capacity, respectively (11, 12, 31). In addition, iPSC-macrophages have been shown to react highly similar to monocyte-derived macrophages to a variety of pathogens (12, 32–34). Similarly, iPSC-derived NK cells show typical NK characteristics and full functionality, as proven in several studies to be able to eradicate HIV-infected CD4^+^ T cells (17), myeloma or pancreatic tumor cells (18) as well as ovarian cancer cells (35). Likewise, iPSC-derived antigen-specific cytotoxic T cells (CTLs) directed against the melanoma epitope MART1 (25) or the WT1 antigen (20), showed antigen-specific reactivity upon stimulation with the respective antigen, proving their functional similarity to *in vivo* CTLs. These protocols are constantly adapted and pave the way for the generation of highly standardized, well-characterized cells from iPSCs, which are derived from healthy or diseased individuals and which can now be used to model IEI *in vitro*. Furthermore, the existence of such differentiation protocols makes these iPSC-derived cell products promising therapeutic agents for “bench to bedside” applications.

## iPSC-based *in vitro* systems to study IEI

The role and importance of iPSC-derived immune cells for the field of IEI is constantly growing and opens new possibilities to study novel forms of treatment. The establishment of numerous disease models for the discovery of the responsible molecular and cellular factors for the clinical phenotype and, at the same time, establishment of promising alternative therapeutic strategies either through the discovery of potent drugs by drug screening approaches or through genetic manipulation of the cells for cell-based therapies, are of great importance. In the chapter below, we cite representative studies for most of the categories of IEI and for which iPSC-derived immune cells have been used to study IEI. A broader overview of the different studies published in this field in the last decade can also be seen in **Table 1**.

**Table 1:** An overview of the latest studies using iPSC-derived cells to study IEI.

	Disease	Gene	Studied cell type (iPSC or iPSC-derived)	Application	Reference
**Combined immunodeficiencies**	SCID	*RAG2*	T cells	Gene Editing	(36)
		*RAG2*	T cells; NK cells	Disease Modeling	(37)
		*RAG1*	T cells	Disease Modeling	(38)
		*JAK3*	T cells	Disease Modeling/Gene editing	(39)
	SCID-X1	*IL-2RG*	NK cells	Gene Editing	(40)
	Reticular dysgenesis	*AK2*	Myeloid; erythroid precursors; myeloid cells	Disease Modeling	(41)
	XLF deficiency	*NHEJ1*	Hematopoietic progenitors	Disease Modeling	(42)
	ADA deficiency	*ADA*	Hematopoietic progenitors; neutrophils	Disease Modeling	(43)
**Combined immunodeficiencies with syndromic features**	AT	*ATM TREX1 RNASEH2B IFIH1*	iPSCs	Drug Screening	(44)
		*ATM*	iPSCs	Gene Editing	(45)
	WAS	*WAS*	Hematopoietic progenitors; T cells; NK cells	Disease Modeling/Gene Editing	(46)
		*WAS*	megakaryocytes	Disease Modeling	(47)
**Predominantly antibody deficiencies**	Hoffman syndrome	*TOP2B*	NK cells	Disease Modeling	(48)
**Diseases of immune dysregulation**	APECED	*AIRE*	iPSCs	Disease Modeling	(49)
	VEO-IBD	*IL10RA* *IL10RB STAT3*	Macrophages	Disease Modeling/Gene Editing/Drug Screening	(50)
**Congenital defects of phagocytes**	SDS	*SBDS*	Hemoangiogenic progenitors; neutrophils; endothelial cells	Disease Modeling	(51)
		*SBDS*	Pancreatic progenitors; mature pancreatic acinar cells; hematopoietic cells	Disease Modeling	(52)
	SCN	*G6PC3*	Granulocytes; neutrophils; monocytes/macrophages	Disease Modeling/Gene Editing/Drug Screening	(53)
		*ELANE*	Granulocytes	Disease Modeling	(54)
		*HAX1*	Myeloid progenitors; neutrophils; monocytes	Disease Modeling/Gene Editing	(55)
	CF	*CFTR*	Lung progenitor cultures	Disease Modeling/Drug Screening	(56)
		*CFTR*	iPSCs	Disease Modeling/Gene Editing	(57)
		*CFTR*	Intestinal epithelia	Gene editing/Drug Screening	(58)
	GATA2 deficiency	*GATA2*	Hemogenic endothelial precursors; hematopoietic progenitors; NK cells	Disease Modeling	(59)
	PAP	*CSF2RA*	Macrophages	Gene Editing	(60)
		*CSF2RA*	Monocytes; macrophages	Gene Editing	(61)
	CGD	*NCF1*	Granulocytes; macrophages	Gene Editing	(62)
		*CYBB*	Granulocytes	Gene Editing	(63)
		*CYBB*	Neutrophils	Gene Editing	(64)
		*CYBB*	Monocytes; macrophages	Gene Editing	(65)
		*CYBB*	Granulocytes	Gene Editing	(66)
		*CYBA* *NCF2*	Neutrophils; macrophages	Disease Modeling	(67)
**Defects in intrinsic and innate immunity**	MYD88 deficiency	*MYD88*	Macrophages	Disease Modeling	(68)
	MSMD	*IFNGR2 IFNGR1 STAT1*	Macrophages	Disease Modeling	(69)
		*IFNGR1*	Macrophages	Disease Modeling	(70)
	TLR3 deficiency	*TLR3*	Trigeminal ganglion neurons	Disease Modeling	(71)
		*TLR3* *UNC93B*	Neural stem cells; neurons; astrocytes; oligodendrocytes	Disease Modeling	(72)
**Auto-inflammatory diseases**	NOMID	*NLRP3*	Monocytes	Drug Screening	(73)
		*NLRP3*	Chondrocytes	Disease Modeling	(74)
	Blau syndrome	*NOD2*	Macrophages	Disease Modeling	(75)
		*NOD2*	Macrophages	Disease Modeling/Gene Editing	(76)
**Bone marrow failure**	FA	*FANCA*	iPSCs; hematopoietic progenitor cells	Disease Modeling	(77)
		*FANCA*	Hemoangiogenic progenitors	Disease Modeling	(78)
		*FANCA*	iPSCs; hematopoietic progenitor cells; mesenchymal stem cells	Disease Modeling/Drug Screening	(79)
	FA-like BMFS	*ADH5 ALDH2*	iPSCs	Disease Modeling	(80)
**Phenocopies of IEI**	NOMID-like disease	*NLRC4*	Macrophages	Disease Modeling	(81)
		*NLRP3*	Macrophages	Disease Modeling/Drug Screening	(82)

Diseases, affected genes, studied iPSC-derived cell types and application (Disease Modeling, Gene Editing, Drug Screening) are summarized. SCID, severe combined immunodeficiency; XLF, XRCC4-like factor; ADA, adenosine deaminase; AT, ataxia-telangiectasia; WAS, Wiskott-Aldrich syndrome; APECED, autoimmune polyendocrinopathy candidiasis ectodermal dystrophy; VEO-IBD, very early onset inflammatory bowel disease; SDS, Shwachman-Diamond syndrome; SCN, severe congenital neutropenia; CF, cystic fibrosis; PAP, pulmonary alveolar proteinosis; CGD, chronic granulomatous disease; MSMD, mendelian susceptibility to mycobacterial disease; NOMID, neonatal-onset multisystem inflammatory disease; FA, Fanconi anemia; FA-like BMFS, Fanconi anemia-like bone marrow failure syndrome.

### Combined immunodeficiencies

One of the most common diseases of this category is severe combined immunodeficiency (SCID), which is characterized by a lack of CD3^+^ T cells. SCID is a life-threatening syndrome with a prevalence of 1/50.000-100.000 worldwide. *IL2RG*, *IL7R*, *JAK3*, *ADA*, *RAG1/2*, and *DCLRE1C* are the most common genes identified to be impaired in SCID patients, resulting in various clinical phenotypes. The current therapeutic approach for SCID patients, apart from antimicrobial drugs, is HSCT partially in combination with gene therapy. The first trial to generate iPSCs from a SCID-patient (adenosine deaminase; ADA deficient-SCID) was conducted in 2008 by Park et al. (83). Later, in 2015 Chang et al. used a patient-specific iPSC line with a mutation in the *JAK3* gene to generate T cells using a two-step OP9 and OP9-DL4 system (39). Studying these iPSC-derived JAK-deficient-T cells showed that JAK deficiency negatively impacts the differentiation of the cells into an early T cell progenitor stage, unraveling the mechanism of immunodeficiency in these patients (39). Correction of the *JAK3* mutation in iPSCs using CRISP/Cas9 technology restored normal T cell development (39). This highlights the importance of iPSC-based *in vitro* systems for studying human lymphopoiesis while developing novel gene correction strategies for human immunodeficiencies at the same time.

ADA deficiency causes abnormal differentiation and function of T cells leading to a severe combined immunodeficiency (84, 85). Recent data from ADA-deficient patients indicated that ADA deficiency impacts myeloid cells, such as neutrophils (43, 86). Given the difficulty in isolating neutrophils from ADA-deficient patients for follow-up studies, using patient-specific iPSCs for generating ADA-deficient neutrophils is very beneficial. Here Tsui et al. could show that ADA-deficient iPSCs generate lower numbers of neutrophils with increased frequency of hyper lobular neutrophils, characterized by decreased phagocytic capacity (43). Thus, the iPSCs technology was able to further associate the contributing mechanisms to the phenotype of ADA-deficient patients (43).

### Combined immunodeficiencies with syndromic features

Ataxia telangiectasia (AT) is an inherited disease characterized by a severe neurological phenotype with a poor prognosis and a lack of efficient accessible treatment. AT is caused by a mutation in the ataxia-telangiectasia mutated gene (*ATM*), leading to a combined immunodeficiency in patients and an increased risk for the development of autoimmunity (87). Not differentiated towards immune cells, iPSCs generated from an AT patient were used as an *in vitro* model to study the cytotoxic effects of the potentially effective immunomodulators thioguanine, mercaptopurine, dexamethasone, mepacrine, thalidomide, and lenalidomide (44). In detail, AT iPSCs were more resistant to thioguanine compared to wild-type iPSCs and at the highest tested concentration of thalidomide and lenalidomide slightly higher cytotoxic effect was observed in AT iPSCs (44). Both AT and wild-type iPSCs were resistant to dexamethasone (44).

As another example, Wiskott-Aldrich syndrome (WAS) is an X-linked inherited immunodeficiency characterized by micro thrombocytopenia, autoimmunity, and hematological malignancies (28). The disease is caused by various mutations in the *WAS* protein gene. Generation of WAS-specific iPSCs and subsequent differentiation to megakaryocytes and platelets contributed to understanding the disease and identifying the responsible molecular and cellular players. More specifically, WAS-iPSC-derived megakaryocytes showed an abnormal pattern of F-actin distribution with abnormal pro-platelet processes, indicating dysregulated cytoskeletal protein rearrangement during pro-platelet formation (47). In this case, the use of patient-derived iPSCs could highlight the importance of the WAS protein for normal platelet production. Similar to the SCID studies, overexpression of the healthy WAS protein in patient- derived iPSCs could rescue the phenotype, paving the way for new therapeutic options. Similarly, Laskowski et al. restored the WAS protein function in patient-derived iPSCs using zinc finger nucleases technology and the differentiated hematopoietic lineages were restored (46). Of note, while differentiation of both healthy and WAS-iPSCs towards non-lymphoid cells was sufficient, a clear reduction in the generation of CD4/CD8 double positive T cells and NK cells was observed, which could be restored upon targeted correction of the *WAS* gene locus.

### Diseases of immune dysregulation

Genetic forms of inflammatory bowel disease (IBD) are caused by mutations in genes that are involved in the IL-10 signaling pathway (88). IBD is characterized by severe bowel inflammation and is developed within the first 6 years of life (89). Many of the patients do not respond to anti-inflammatory and immunosuppressive treatments. To study the pathophysiology of IBD and contribute to novel therapeutic strategies, KO iPSC models for the genes *IL10R*, *IL10RB*, *STAT1*, and *STAT3* were generated using sgRNA-directed CRISPR-Cas9 lentiviral vectors (50). Using macrophages derived from these KO-iPSC lines these studies could show that defects in any of the IL10R chains or in STAT3 result in absence of BCL3 expression and reduced secretion of defined IL-10R-/STAT3-dependent cytokines (50). Of note, the phenotype of the KO-iPSC-derived macrophages could however be restored (reduced pro-inflammatory cytokines) using lentiviral vectors overexpressing the *IL-10R* gene. Using the same iPSC-macrophage system, small anti-inflammatory agents (SB202190 and Filgotinib) were tested and could confirm their anti-inflammatory effect by the reduction of TNF-α, IL-6, and CCL5, while no negative impact could be observed on iPSC-derived macrophages with respect to cell viability (50).

### Congenital defects of phagocytes

Chronic granulomatous disease (CGD) is characterized by severe, recurrent, and life-threatening bacterial and fungal infections due to defects in the oxidative burst in phagocytes. Its prevalence is 1/250,000 individuals. CGD can be caused by mutations in any of the four components of the NADPH oxidase complex. The most common mutation is found in the *CYBB* gene, which encodes for the gp91^phox^ subunit and is X-linked. To date, the only available treatment is allogeneic or autologous (genetic corrected) HSCT. To elaborate on new treatments for X-CGD patients, several studies have used X-CGD-patient-specific iPSCs to genetically modify the cells using zing finger nuclease-mediated gene targeting (90), transcription activator-like effector nucleases (TALENs) (66) or bacterial artificial chromosomes (BAC) transgenesis (64), respectively. In all iPSC-based studies, the *CYBB* function was successfully restored, leading to sufficient oxidative activity and ROS production in iPSC-derived granulocytes, proven the suitability of gene therapy to restore the anti-microbial function in immune cells. In a completely different approach, the NADPH oxidase activity of X-CGD iPSC-derived macrophages was restored using NOX2/p22^phox^ proteoliposomes, which were transported into the macrophages (91). The combination of patient-specific iPSC-derived cells with recombinant therapeutic proteoliposomes could in the future lead to the development of alternative antibacterial or antifungal therapies for patients with IEI. Similar to the aforementioned approaches, the iPSC system has also been used to establish and test gene correction of p47^phox^ deficiency. Introducing a functional *NCF1* minigene into the intron 1 of the *NCF1* gene using CRISPR/Cas9 (62) or targeted correction of the mutation (GT deletion in NCF1 pseudogenes) using zinc-finger nucleases (92) into p47-CGD iPSCs could restore oxidase function in iPSC-derived immune cells, highlighting the suitability of the iPSC system to test novel gene therapy concepts.

### Defects in intrinsic and innate immunity

Mendelian susceptibility to mycobacterial disease (MSMD) is characterized by increased susceptibility to weakly virulent mycobacteria (e.g. *Mycobacterium bovis* Bacillus Calmette-Guerin; BCG). The genetic etiology of MSMD is complex, with a variety of genes and mutations involved, which all affect the sufficient breakdown of mycobacteria. Mutations can affect either T cells (e.g. *IL12RB1*, *IL12RB2*, *TYK2*) or macrophages (e.g. *IFNGR1*, *INFGR2*, *IRF8*, *CYBB*, *NEMO*), which lead to an impaired crosstalk of these two cell types. As an example, the clinical phenotype can be severe, as seen in patients with complete IFN-gamma receptor 1 or 2 deficiency (*IFNGR1/2)*. In contrast, the clinical phenotype can also be mild to moderate, as seen in patients suffering from STAT1, IL-12/IL-23 receptor, or tyrosine kinase 2 deficiency. Of note, clinical symptoms and the impaired function of e.g. macrophages can be improved by treating patients with high dose IFNγ therapy. However, this kind of treatment is unsuitable for patients who suffer from complete *IFNGR1* or *IFNGR2* deficiency. The generation of iPSCs from patients harboring mutations in genes involved in the IFNγ signaling, such as *IFNGR1*, *IFNGR2*, and also *STAT1* were able to demonstrate in detail the impact of IFNγ on macrophages and the importance of this cell type in the onset and progression of mycobacterial susceptibility (69, 70). These studies revealed iPSC-derived macrophages with an impaired type II IFN system showing normal macrophage differentiation and phenotype but severely impaired intracellular killing activity for BCG (69, 70).

### Autoinflammatory diseases

Neonatal-onset multisystem inflammatory disease (NOMID), also known as chronic infantile neurologic cutaneous articular syndrome (CINCA), is a rare genetic disease present from birth and caused by mutations mainly in the *NLRP3* locus. It is inherited in an autosomal dominant way, and the patients suffer from uncontrolled inflammation in several systems of the body, such as skin, joints, and central nervous system. The clinical phenotype varies and includes urticarial-like skin rash, arthritis, and chronic meningitis, which increases the risk of neurological problems. So far, anti-IL-1β treatment (e.g. Anakinra) using specific inhibitors is the preferable therapeutic option. However, its effectiveness is highly dependent on the severity of the disease phenotype. In addition, the complete IL-1β blockade involves the risk of uncontrolled immunosuppression. For this reason, selective NLRP3 inhibitors would be more beneficial as therapeutic option and several NLRP3 inhibitors have already entered clinical trials (93). However, given the different mutations observed in NOMID patients, it is possible that some NLRP3 mutants escape an efficient inhibition from already known inhibitors (94). Therefore, discovery of novel NLRP3 inhibitors is necessary. To test and screen for new therapeutic compounds, patient-specific NOMID-iPSCs could be used as a screening platform. Seki et al. generated iPSC-derived immortalized myeloid cell lines from wild-type and NLRP3-mutated iPSC clones and subsequently differentiated these into macrophages (73). Generated macrophages were further used for developing a high throughput system to identify compounds that show inhibitory effects specifically against the secretion of IL-1β and the activation of mutant NLRP3 (73). Out of almost 5,000 tested compounds, seven candidates were sufficiently blocking the IL-1β secretion. Interestingly those were already introduced in previous studies as NLRP3 inhibitors, indicating the effectiveness of the system (73).

### Complement deficiencies

In addition to increased susceptibility and recurrent bacterial infections, deficiencies in the complement pathway have been linked to age-related macular degeneration (AMD). AMD is an ideal example underlining the interconnection of IEI with various tissues and organs of the body. Thus, using iPSCs generated from patients with AMD or from healthy individuals shed light on the mechanisms involved in the disease and showed the impact of an IEI in a complement protein on the progression of AMD. In this case, retinal pigment epithelium derived from AMD-derived iPSCs was used to show impaired mitochondrial function under stress conditions and its link to the presence of the high-risk allele for the complement factor H (*CFH* locus) (95). More generated iPSC lines from three patients carrying the rare variants in the *CFH* locus and suffering from AMD are available tools for further cellular studies and the development of novel treatments (96).

### Bone marrow failure

Fanconi anemia (FA) is an inherited condition diagnosed usually in children between the age of 3 and 14, and it is characterized, among other symptoms, by failure of bone marrow function. It is caused by mutations in at least 22 different genes, which are involved in the FA pathway, responsible for the DNA repair process, with the genes *FANCA*, *FANCC*, and *FANCG* to be utmost affected. The only curative treatment so far is HSCT, with gene therapy on the horizon (97). Given the difficulty that exists in recapitulating FA pathophysiology using mouse models, further understanding of the disease pathogenesis was accomplished using FA patient-specific iPSCs for disease modelling (77, 79). Marion et al. revealed that activation of the p53-p21 axis leads to accelerated erythroid differentiation in FANCA-deficient HPCs. Use of exogenous recombinant human GAS6 resulted in restored hematopoiesis, providing alternative options for improving therapy of FA in the future (77). In contrast, another study utilized the iPSC technology to screen and evaluate novel compounds, discovering that Tremulacin was able to rescue the hematopoietic defects of FA patient by suppressing the transcription of the inflammatory cytokine TNFα (79), which impressively shows the potential of the iPSC technology.

## Current limitations and future perspectives

The numerous studies that have used iPSC-derived cells for disease modeling and drug screening for several IEI-related conditions could lay the foundation for developing novel therapies. The reason for this success is the unique potential of iPSCs to differentiate into almost all cells of the hematopoietic system and beyond. Although established protocols for the differentiation of a plethora of iPSC-derived immune cells exist, still the lack of protocols for a robust generation of T or B lymphocytes that resemble their *in vivo* counterparts as much as possible, is currently a limitation of the technology and must be confronted in the coming years.

While most of the aforementioned reports have used iPSCs as a disease modeling platform, the use of iPSC-based platforms for establishing drug screening, drug toxicology, and drug-drug interactions in the context of IEI is highly warranted and will accelerate the progress of personalized medicine further. Along this line, several studies used iPSC-derived cells for targeted drug testing or screening (44, 50, 53, 56, 58, 79). However, these attempts did not result in developing a widely accepted drug so far. Consequently, researchers have not fully exploited the full potential of iPSCs until now, and novel approaches are currently underway to solve this issue. Besides drug discovery in the context of IEI, the further improvement of existing differentiation protocols from feeder-based GMP-incompatible systems to xeno-free and GMP-compatible protocols, drove iPSC-derived cells to the first clinical trials. In 2021, 19 therapeutic clinical studies were globally ongoing (98). Interestingly, none of them was related to IEI, highlighting the effort that should be invested in the next years to bring the undoubtable benefits that we can gain from the iPSC technology closer to the clinic. One of the main challenges in using iPSC-derived cells as a cell-based therapeutic intervention are the immunohistocompatibility issues arising from the use of allogeneic cells. Most clinical trials currently use allogeneic cells since the generation of autologous iPSCs is time-consuming, which becomes a particular issue when the recipient urgently needs cell therapy. Therefore several studies have tried to elaborate alternative options, either by developing cell banks with homozygous iPSC lines or by generating immunocompatible iPSCs through genetic manipulation (99–101).

Given the very young age of most patients with IEI, the elimination of the possible tumorigenicity of iPSCs and iPSC-derived cells, should be highly warranted, before the use of iPSC-derived cells for the treatment of children suffering from IEI. In order to diminish the impact of the integrating vectors on the iPSCs genetic stability and to provide a reliable tool to generate novel therapies against IEI, different reprogramming strategies have been developed, such as the use of non-integrating vectors, synthetic mRNAs, or integrating vectors that can be excised. Establishing a universal and highly standardized procedure for confirming the genetic stability and purity of iPSCs to get approved for clinical use, could be very beneficial. Usage of Next Generation Sequencing analysis should also be considered to guarantee the detection of all possible genetic anomalies and to ensure the production of high-quality iPSCs with limited risk for tumorigenicity (102). To further minimize the tumorigenic risk, in the last two decades, several systems have been developed for the elimination of aberrant cells using suicide gene technology (103–108). Most recently, immunodepletion has also been used to selectively deplete contaminating iPSCs with the help of monoclonal antibodies (109, 110) or chimerized monoclonal antibodies (111). Further optimization of these tools will significantly assist in facilitating the safe use of iPSC-derived cells in the clinical setting in the future.

While iPSC and thereof derived cells are used frequently for modeling IEI the clinical translation of cells to treat IEI is more in the future.

## Authors contribution

EN, JR, GH, and NL designed, wrote and approved the manuscript. All authors contributed to the article and approved the submitted version.

## Funding

This project has received funding from the European Research Council (ERC) under the European Union’s Horizon 2020 research and innovation program (grant agreement No. 852178). The work is also funded by the Deutsche Forschungsgemeinschaft (DFG, German Research Foundation) under Germany’s Excellence Strategy - EXC 2155 - project number 390874280 and REBIRTH Research Center for Translational Regenerative Medicine “Förderung aus Mitteln des Niedersächsischen Vorab” (grant: ZN3340).

## Acknowledgments

We thank Shifaa Abdin and Mania Ackermann for critical review of the manuscript. The figure has been created with BioRender.com.

## Conflict of interest

NL filed and licensed patents in the field of iPSC-derived macrophages outside of the MS. NL is a consultant for CATALENT outside of the MS.

The remaining authors declare that the research was conducted in the absence of any commercial or financial relationships that could be construed as a potential conflict of interest.

## Publisher’s note

All claims expressed in this article are solely those of the authors and do not necessarily represent those of their affiliated organizations, or those of the publisher, the editors and the reviewers. Any product that may be evaluated in this article, or claim that may be made by its manufacturer, is not guaranteed or endorsed by the publisher.
